# The effect of ambient air pollution on respiratory health of school children: a panel study

**DOI:** 10.1186/1476-069X-7-16

**Published:** 2008-05-14

**Authors:** Michael J Epton, Robin D Dawson, Wendy M Brooks, Simon Kingham, Teresa Aberkane, Jo-Anne E Cavanagh, Christopher M Frampton, Tracey Hewitt, Julie M Cook, Susan McLeod, Fiona McCartin, Katherine Trought, Leslie Brown

**Affiliations:** 1 Canterbury Respiratory Research Group, Department of Medicine, Christchurch School of Medicine and Health Sciences, University of Otago, PO Box 4345, Christchurch 8140, New Zealand; 2 Department of Geography, University of Canterbury, Private Bag 4800, Christchurch 8020, New Zealand; 3 Environment Canterbury, 58 Kilmore Street, Christchurch, New Zealand; 4 Landcare Research, P O Box 40, Lincoln 7640, Christchurch, New Zealand

## Abstract

**Background:**

Adverse respiratory effects of particulate air pollution have been identified by epidemiological studies. We aimed to examine the health effects of ambient particulate air pollution from wood burning on school-age students in Christchurch, New Zealand, and to explore the utility of urine and exhaled breath condensate biomarkers of exposure in this population.

**Methods:**

A panel study of 93 male students (26 with asthma) living in the boarding house of a metropolitan school was undertaken in the winter of 2004. Indoor and outdoor pollution data was continuously monitored. Longitudinal assessment of lung function (FEV_1 _and peak flow) and symptoms were undertaken, with event studies of high pollution on biomarkers of exposure (urinary 1-hydroxypyrene) and effect (exhaled breath condensate (EBC) pH and hydrogen peroxide concentration).

**Results:**

Peak levels of air pollution were associated with small but statistically significant effects on lung function in the asthmatic students, but not healthy students. No significant effect of pollution could be seen either on airway inflammation and oxidative stress either in healthy students or students with asthma. Minor increases in respiratory symptoms were associated with high pollution exposure. Urinary 1-hydroxypyrene levels were raised in association with pollution events by comparison with low pollution control days.

**Conclusion:**

There is no significant effect of ambient wood-smoke particulate air pollution on lung function of healthy school-aged students, but a small effect on respiratory symptoms. Asthmatic students show small effects of peak pollution levels on lung function. Urinary 1-hydroxypyrene shows potential as a biomarker of exposure to wood smoke in this population; however measurement of EBC pH and hydrogen peroxide appears not to be useful for assessment of population health effects of air pollution.

Some of the data presented in this paper has previously been published in Kingham and co-workers Atmospheric Environment, 2006 Jan; 40: 338–347 (details of pollution exposure), and Cavanagh and co-workers Sci Total Environ. 2007 Mar 1;374(1):51-9 (urine hydroxypyrene data).

## Background

Epidemiological studies have established the adverse health effects of particulate air pollution, particularly in relation to cardiac and respiratory effects [1,2]. However, direct assessment of health impacts of air pollution on individuals presents significant challenges, including the difficulty and expense of accurately assessing personal exposures [3,4], variation in exposure in home versus school/work and outdoor versus indoor environments and exposure to tobacco smoke. The source of the particulate pollution may also be relevant. The majority of research from the USA and Europe has studied particulate pollution from coal-fired power stations, heavy industry, and diesel engines. However, wood and other organic matter combustion may be significant source of exposure in areas burning solid fuel for domestic heating, such as Canada and the North West USA, Scandinavia, and New Zealand. Indeed there is an increasing trend for wood to be used as a source of home heating in the New England states and in upstate New York.

Direct assessment of lung effects has generally been limited to measurement of peak expiratory flow rate (PEFR) diaries, which, like any self-administered test involving writing down numerical values, may be prone to subjective bias, end-digit preference and auto-correlation. More recently, the availability of electronic downloadable spirometers means detailed lung function assessment in large cohorts can be undertaken easily and cheaply. The spirometers incorporate quality assessment protocols and provide a more objective assessment of lung function. In addition, techniques such as exhaled breath condensate collection now allow non-invasive assessment of airway inflammation, and potentially provide information about oxidative stress responses in the lungs.

The aim of this study was to examine the health effects of ambient particulate air pollution (largely derived from wood-burning for home heating) on school-age children in a panel study design, and to explore the utility of urine and exhaled breath condensate biomarkers of exposure in this population. To reduce exposure variation, and provide accurate assessment of exposure, school children from the boarding houses of a metropolitan private school were studied. Longitudinal studies of lung function effects were undertaken using personal electronic spirometers and symptom diaries, and, after high pollution events, detailed event studies of pollution effects were undertaken using exhaled breath condensate and urine collection.

Some of the data presented in this paper has previously been published in Kingham and co-workers (details of pollution exposure) [5] and Cavanagh and co-workers (urine hydroxypyrene data) [6]

## Methods

### Study site

The study was undertaken in winter 2004 in Christchurch, New Zealand, a city of ~330,000 situated on the east coast of the South Island. Significant air pollution in Christchurch almost invariably occurs in winter, and is characterized by PM_10_, ~90% of which is PM_2.5_, that is largely generated by domestic wood-burning open fires and enclosed wood-burners [7]. Mean daily PM_10 _in central and suburban Christchurch exceeds 50 μg/m^3 ^on ~30 days per year and can reach up to 250 μg/m^3 ^[8]. The health effects of Christchurch air pollution have been the subject of a number of local studies [9-11].

The study was undertaken in the boarding houses at Christ's College, a single-sex private school situated in the central city, but inset into the grounds of Hagley Park, a five km^2 ^area of grassland and trees to the immediate west of the CBD but surrounded by the urban area on all sides.

### Pollution monitoring

Two pollution monitoring sites were established for this project. The indoor site was a second floor corridor leading to rooms occupied by boarding students. The building is naturally ventilated and is heated by a thermostatically controlled central heating system. The site was not near any obvious indoor sources of particulates such as kitchens which are located in a different building. The outdoor site was a courtyard within the school grounds. Indoor and outdoor particulate pollution levels (PM_10_, PM_2.5_, PM_1_) were measured continuously using a TEOM Series 1400a Ambient Particulate Monitor, and expressed as 10 minute averages over a 24 hour period, and 24 hour averages, from midday to midday. It has been shown that this TEOM under-reads the true PM_10 _mass because of the loss of semi-volatile compounds in the heated air stream. Consequently the figures were corrected based on an established factor. Details about this and the more information about the indoor and outdoor pollution monitoring at Christ's College are given in Kingham and co-workers [5]. Weather data including wind speed, temperature, temperature difference and relative humidity were also continuously collected from the outdoor site and expressed as 10 minute averages.

### Participants

The participants were male secondary school students, aged between 12 and 18 years, attending the boarding houses of Christ's College. All were healthy non-smokers with no significant medical conditions. Smokers were specifically requested not to volunteer for the study, and the non-smoking status of the participants was confirmed by school staff. A sub-group of students with a previous diagnosis of asthma (defined as "doctor diagnosed asthma ever") were also studied. They were being treated with as-required short-acting beta agonist reliever medication, with some taking regular inhaled corticosteroids (see Results section for details). The regular use of oral corticosteroids was an exclusion criterion. Investigators made no alterations to asthma management and gave no advice about reliever inhaler use during the study.

Prior to commencing the study the health status of the students was assessed by taking a brief medical history. Baseline spirometry and skin prick testing for atopic status were also undertaken.

The study protocol and conduct was approved by the Canterbury Regional Ethics committee. All participants provided written informed consent prior to commencement, and parental approval to participate was also sought.

### Lung function assessment

All students were issued with personal downloadable electronic spirometers (Piko-1, Ferraris Respiratory Europe Ltd, Hertford, UK) and trained in their use. These spirometers measure Forced Expiratory Volume in 1 second (FEV_1_) and peak expiratory flow rate (PEFR) to American Thoracic Society standards [12]. Lung function measurement was undertaken twice daily, in the early morning, and around 6.00 pm, supervised by House Matrons, who were all trained nurses given training in spirometry. No specific instructions about withholding reliever medications prior to spirometry were given to the students. Spirometry data were downloaded at least fortnightly by investigators.

### Diary cards

All students were issued with daily diary cards to detail respiratory, nasal and eye symptoms. In addition, students with asthma completed diaries detailing asthma symptoms and reliever medication use.

Lung function and diary card data collection occurred during school terms for the duration of the project. When a student was away from the school for more than one night (weekend and school holidays), diaries and lung function measurements were not undertaken.

### High pollution peak assessment

Within 24 hours of two separate high pollution days all participating students underwent a more detailed assessment. The investigators were informed by Environment Canterbury the morning after a high pollution night. The school was contacted, and all participating boys were excused their evening study activities, to be assessed by the investigating team. This involved collection of urine samples for analysis of 1-hydroxypyrene and collection of exhaled breath condensate for measurement of pH and hydrogen peroxide. Baseline assessments were also undertaken at the beginning of the study during a period of low pollution (autumn control) and during the winter, after a 1 week period of low pollution levels (winter control).

### Urinary 1-hydroxypyrene levels (1-OHP)

Urine samples were stored at -20°C until analysis. Urinary 1-OHP was quantified using reverse phase high-performance liquid chromatography (HPLC) using a modification of a previously described method [13] as described in Cavanagh and co-workers [6]. The lower limit of detection was 0.23 nmol/L. Hydroxypyrene concentrations were corrected for urinary creatinine to account for hydration status and expressed as μmol 1-OHP/mol creatinine.

### Exhaled breath condensate (EBC) collection

Exhaled breath condensate (EBC) was collected using the RTube™ system (Respiratory Research Inc, Charlottesville, USA), using an aluminium cooling tube. The aluminium cooling tube was stored at -20°C for at least 24 hours prior to collection. EBC was collected for ten minutes on each sampling occasion, after rinsing the mouth to reduce saliva contamination. Approximately 2 ml of condensate was collected from each student at each collection.

### EBC pH

EBC pH was immediately measured by applying approximately 30 μl of EBC to a pre-calibrated Shindengen ISFET pH meter (model KS701). EBC was not de-gassed prior to pH measurement.

### EBC hydrogen peroxide

H_2_O_2 _was measured in EBC samples using fluorometry with 4-hydroxyphenylacetic acid [14]. Briefly, following the H_2_O_2_-dependent conversion of 4-HPAA to the dimer 2,2'-dihydroxybiphenyl-5,5'-diacetate, the fluorescence of each sample was quantified within four hours using a Hitachi F4500 Fluorescence Spectrophotometer (excitation 295 nm, emission 405 nm; slit width 5 nm ex/10 nm em; PMT voltage 700 V). Linear regression analysis allowed the estimation of the concentration of H_2_O_2 _in samples based upon a corresponding standard curve. More details of the H_2_O_2 _assay as used in our laboratory, including validation and optimisation can be found in Brooks and co-workers [15]. The limit of detection of the assay used for these experiments was 3.4 nM H_2_O_2_.

### Statistics and sample size

For seasonal and event data comparisons of EBC pH, hydrogen peroxide, and 1-OHP concentrations were compared using the non-parametric Friedman's test and Wilcoxon-signed rank tests as appropriate to allow for repeated observations on individuals and due to the extreme non-normality of these measures. Readings below the limit of detection for any assay were arbitrarily assigned a value corresponding to 50% of the limit of detection to avoid division by zero in any subsequent calculation. Longitudinal data was cleaned to exclude spurious or physiologically impossible data points by an *a priori *defined protocol. Peak flow and FEV_1 _data for each student was expressed as a Z-score relative to the student's mean score over the period of the study. Longitudinal data analysis exploring bivariate association between lung function (Z-scores of PEFR or FEV_1_) or symptom data and pooled air pollution measures (both indoor and outdoor) and climate related measures were conducted using the non-parametric Spearman's correlation coefficient. The air pollution measures were lagged daily out to seven days and correlated with lung function and symptom scores. In this manner the potential for any lagged effect of air pollution on these measures was analysed. Variables showing some association (p < 0.15) with lung function or symptom data from these analyses were then entered into a mixed-model linear regression analysis which included the daily use of a reliever as a covariate to confirm the independent association with lung function or symptoms. These analyses were undertaken for the dataset as a whole and repeated separately for asthmatic and non-asthmatic students.

In addition, mean levels of each of the longitudinal outcome variables for each student were calculated within moderate/high (24 hour average> 20 μm/m^3 ^outdoor) pollution days and low pollution days. These levels were compared using Mann-Whitney U tests. Longitudinal data analysis exploring bivariate association between lung function (Z-scores of PEFR or FEV_1_) and pooled air pollution measures on moderate/high pollution (24 hour average> 20 μm/m^3 ^outdoor) days alone were then conducted using the non-parametric Spearman's correlation coefficient.

The power of the study was of necessity dependant on the number and magnitude of high pollution days during the study period. In addition, limiting the sampling to one school (to increase exposure validity) placed limitations on potential numbers of students willing/able to participate. Retrospective power analysis was therefore undertaken for lung function scores (expressed as Z-scores) between moderate/high (24 hour average> 20 μm/m^3 ^outdoor) pollution days and low pollution days. Assuming a 2:1 split in air pollution days, and given n = 80 students, the study had an 80% power to detect differences in Z-score values of 0.30 in FEV_1 _and 0.27 in PEFR with α = 0.05 (two-tailed). In addition, with 100 time points a correlation between Z-scores and pollution of >0.28 would have been detected as statistically significant i.e. R^2 ^>10%.

## Results

### Students' baseline demographics and lung function

A total of 93 students took part in the study, from a total potential boarding house population of 240 students. Eighty-nine students identified themselves as New Zealand European, whilst four (4.3%) came from Asia. There were no students who identified themselves as Maori. In 2001, 6.9% of the population of Christchurch identified themselves as Maori, while 5.5% identified themselves as Asian [16]. Twenty-six (28%) of the students had a doctor diagnosis of asthma. The prevalence of asthma in this age group in New Zealand is 24.4% [17]. Seventeen of these students used short-acting beta agonists on an as-required basis, while eight students were taking regular inhaler corticosteroids. Mean age of the students at study start was 14 years 5 months (range 12 years 9 months – 17 years 6 months). There were no significant age differences between students with asthma and those without asthma. There was no significant difference in % predicted FEV_1 _between asthmatics and non-asthmatics (97.54% ± 2.481 (mean ± 1 s.e.) n = 26, vs 95.49% ± 1.635 n = 57), though FEV_1_/FVC ratio was significantly lower in the asthmatic students (FEV_1_/FVC ratio asthma group 0.803 ± 0.013 n = 26 vs normal group 0.834 ± 0.009 n = 57, p = 0.05).

### Asthmatic reliever use

Reliever use in the asthmatic students was generally low. Reliever use was only documented on 3.26% of the days in the diary cards filled in by the asthmatic students. There was no significant difference between the number of puffs of reliever used by the asthmatics on low pollution days when compared to moderate/high days (Mann-Whitney U, p = 0.488). There was however a relationship between days of ANY reliever use and the occurrence of a moderate/high pollution day. For this reason, the daily use of a reliever was used as a covariate in the mixed-model linear regression analysis.

### Air pollution

**Figure 1:**
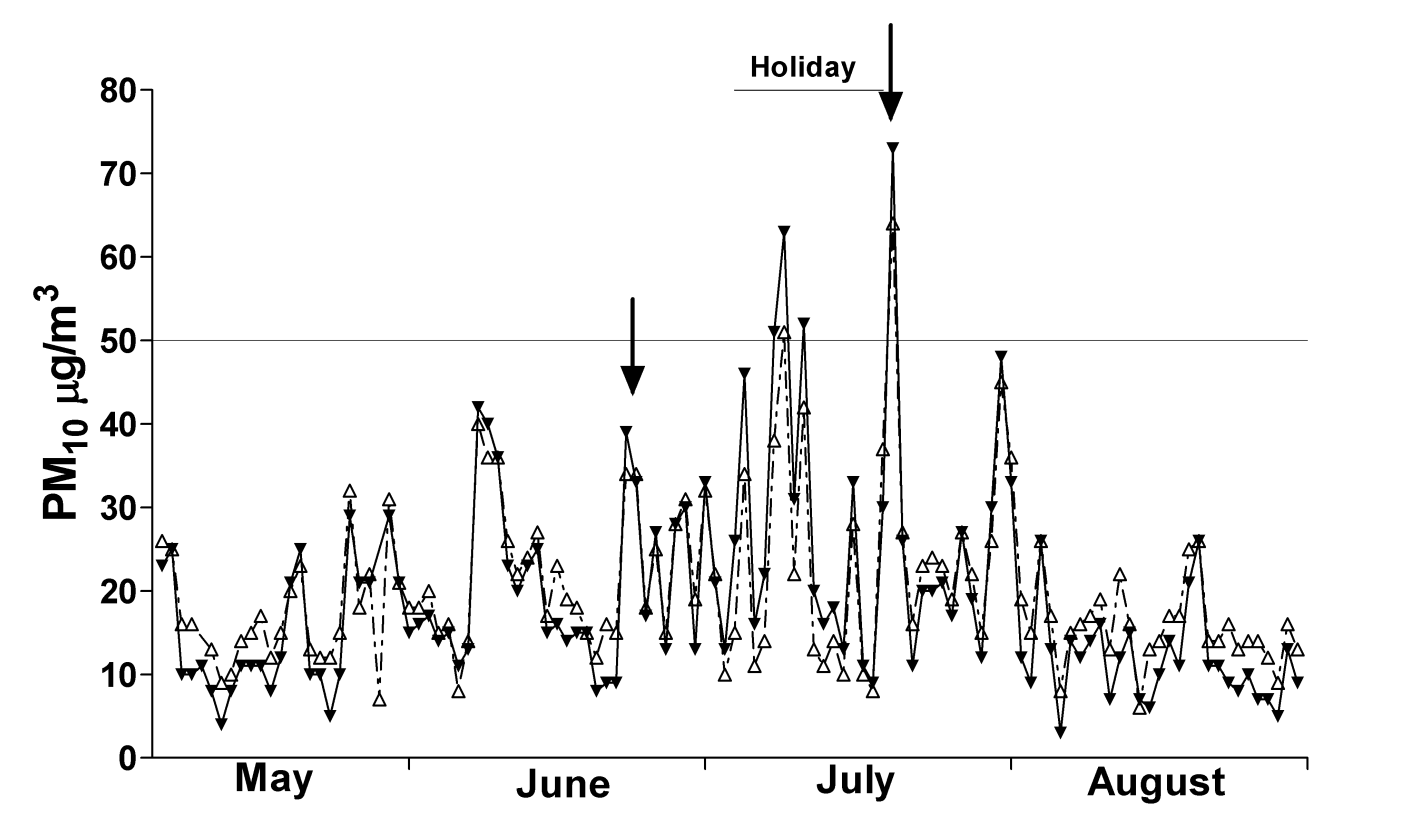
**Indoor (open triangle) and outdoor (closed triangle) 24 hour mean PM_10 _levels**. The school holiday period, when no biological data was collected, is marked. Detailed event studies occurred after high pollution nights marked with arrows. The New Zealand National Environmental Standard cut-off for high pollution days is marked at 50 μg/m^3^. Details of monitoring sites and positions are given in the Methods section.

**Figure 2:**
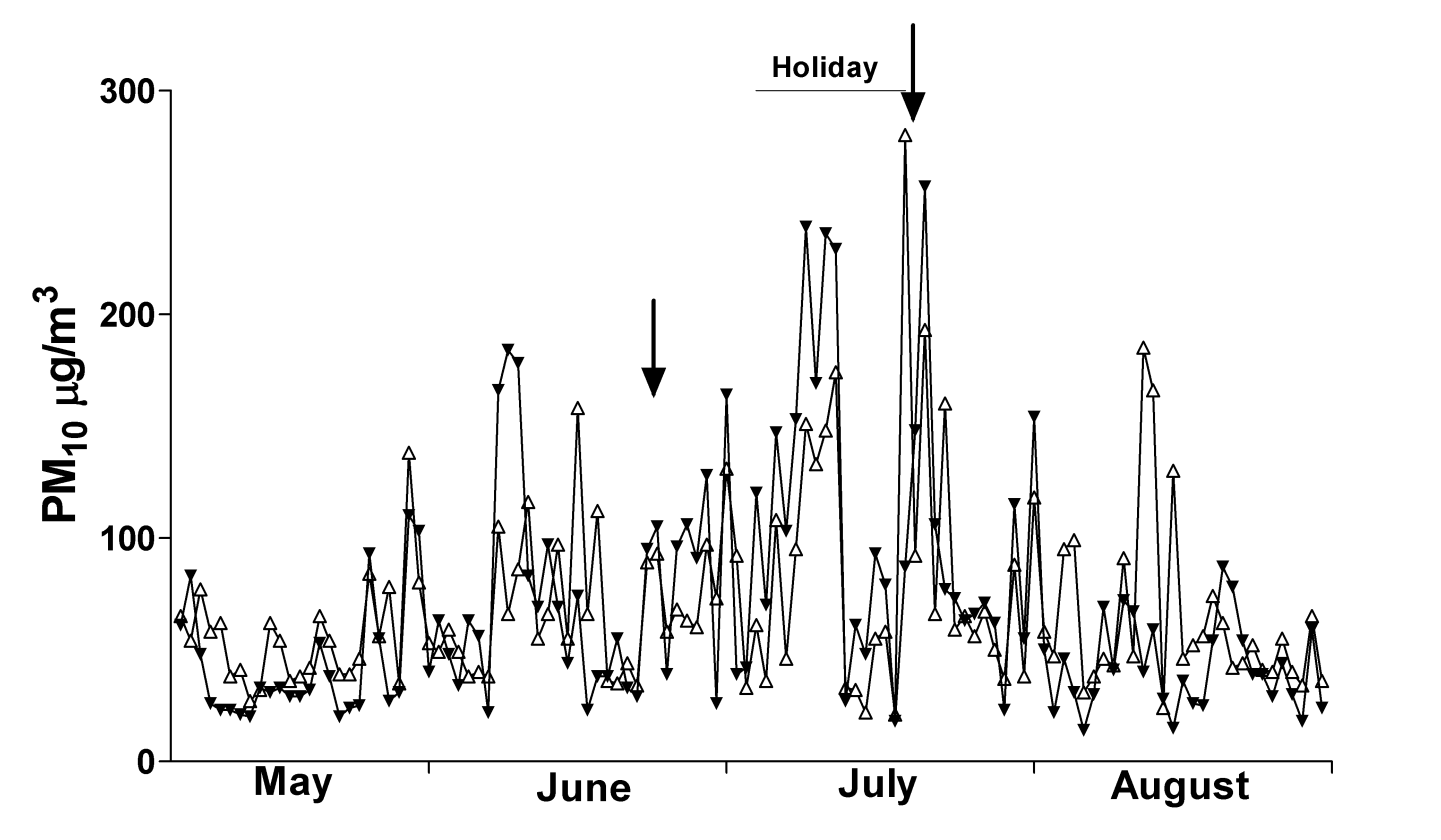
**Indoor (open triangle) and outdoor (closed triangle) 10-minute peak PM_10 _levels**. The school holiday period, when no biological data was collected, is marked. Detailed event studies occurred after high pollution nights marked with arrows. Details of monitoring sites and positions are given in the Methods section.

The study was undertaken from the end of March to early September 2004, encompassing autumn and winter. Indoor and outdoor pollution levels are shown in Figure 1 (24 hour average) and Figure 2 (10-min peak). Pollution levels during the winter of 2004 were generally lower than previous years [18], with the majority of high pollution nights occurring during the school vacation period, when data was not being collected. There were however a number of high pollution events where data was collected. Detailed sampling took place after high pollution nights on June 24 and July 21. The 24-hour average outside PM_10 _level recorded at the school on those dates was 43 μg/m^3 ^and 72 μg/m^3 ^respectively. Peak outdoor PM_10 _levels for a 10-minute period reached 105 μg/m^3 ^and 257 μg/m^3 ^respectively. The majority of PM_10 _pollution was in the PM_2.5 _range, and indoor pollution levels were similar to outdoor. Occasional very high pollution peaks were identified indoors. These tended to be in the PM_10 _range, rather than PM_2.5_, and probably represented resuspension events due to activity in corridors. On average, pollution levels measured at the school were 22.5% lower than those measured at the central Environment Canterbury monitoring station.

### Urinary 1-OHP levels

**Figure 3:**
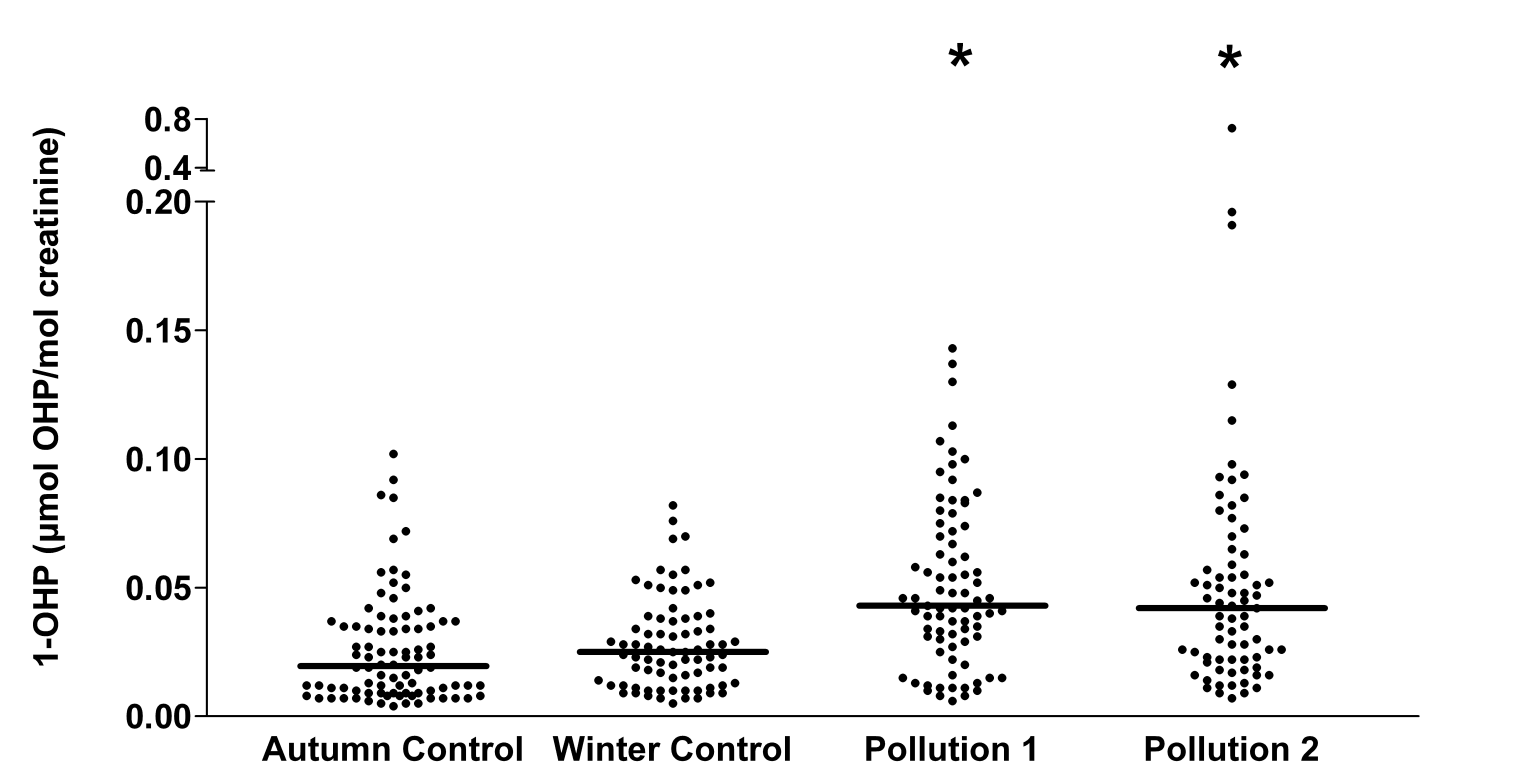
**Urinary 1-hydroxypyrene levels (adjusted for creatinine) on the control and high pollution assessment days**. * p < 0.0001 for all differences between high pollution days and controls. Data in this graph is also shown in graph form in Cavanagh and co-workers [6].

Urine 1-OHP levels are shown in Figure 3. Urine 1-OHP levels were significantly higher on the high pollution days compared to both low pollution control days. Median (25–75% range, number of sample analysed) 1-OHP levels corrected for creatinine were: Autumn control 0.0195 (0.009–0.036, n = 88) μmol OHP/mol creatinine; Winter control 0.025 (0.013–0.038, n = 77) μmol OHP/mol creatinine; Pollution day 1 0.043 (0.030–0.073, n = 79) μmol OHP/mol creatinine; Pollution day 2 0.042 (0.022–0.064, n = 73); p < 0.0001 for all comparisons of high pollution days vs controls. Differences in 1-OHP levels between the two control days did not reach statistical significance. Similarly, the 1-OHP levels on the two high pollution days were not significantly different.

### EBC pH

**Figure 4:**
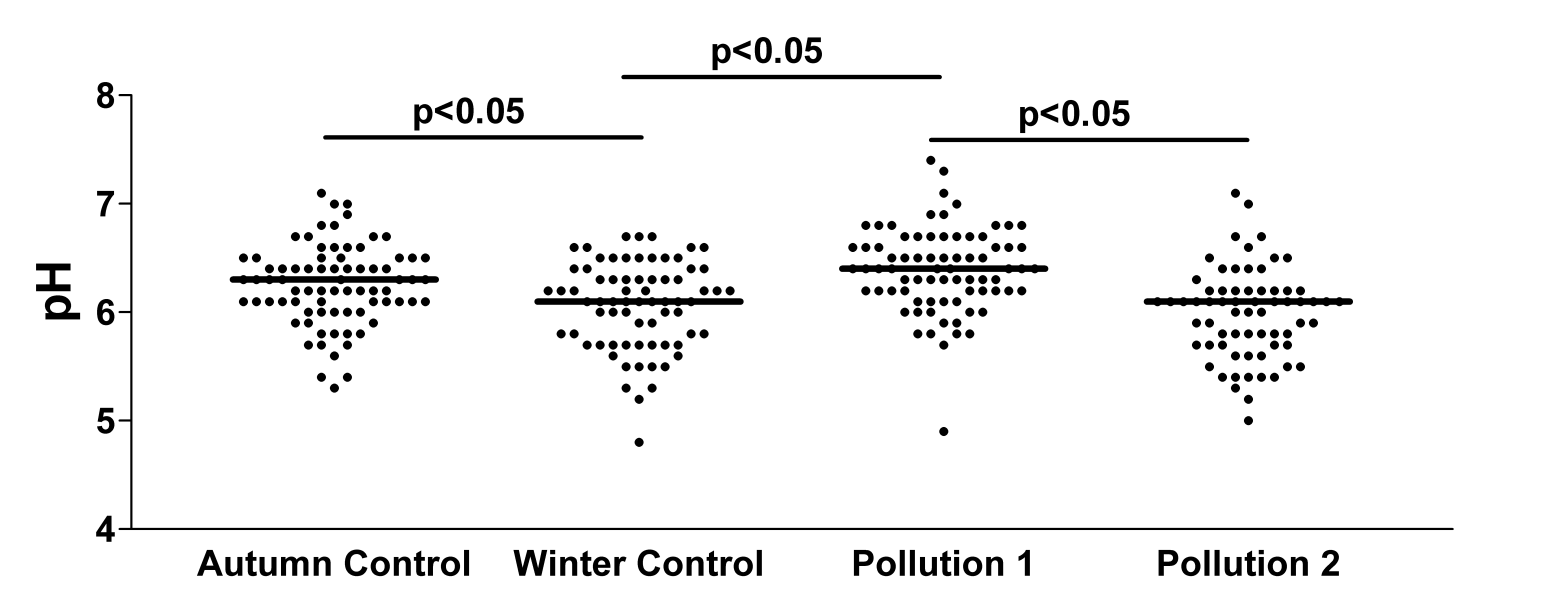
Exhaled breath condensate pH levels on the control and high pollution assessment days.

EBC pH readings are shown in Figure 4. There was a broad range of pH readings on all sampling days. pH differences between autumn and winter controls reached statistical significance, and pH at the first high pollution day was significantly higher than the second study day, and the winter control day, but not the autumn control day. There was no independent effect in students with asthma in comparison with healthy students.

### EBC hydrogen peroxide

**Figure 5:**
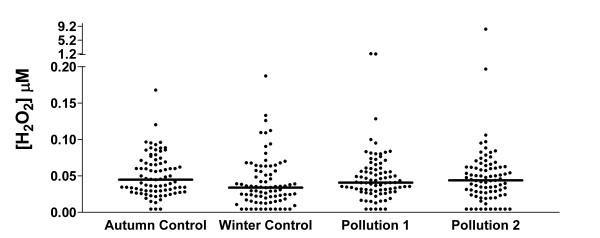
Exhaled breath condensate H_2_O_2 _levels on the control and high pollution assessment days.

EBC H_2_O_2 _readings are shown in Figure 5. Detectable hydrogen peroxide was present in most samples. There was no difference in measured H_2_O_2 _between any of the sampling days, either control or high pollution. There was also no difference in proportions of samples with detectable H_2_O_2_. There was no independent effect of asthma in comparison with healthy subjects. There was no significant correlation between EBC levels of hydrogen peroxide, and pH, and no correlation with either of these measurements and urinary 1-OHP.

### Lung function data

FEV_1 _data was available for a median of 129.5 time points per student (Range 4–189). PEFR data was available for a median of 128 time points (Range 2–186). Univariate analysis identified a number of biologically plausible correlations between lung function data and PM_10 _levels which reached statistical significance. There was a significant correlation between morning FEV_1 _and 24 hour average outdoor air pollution levels the previous day (Spearman's rho -0.201, p = 0.034). When analysing for the presence of asthma, asthmatic subjects showed correlations between FEV_1 _and 24 hour average outdoor air pollution levels which just failed to reach statistical significance (Spearman's rho -0.187, p = 0.06), while non-asthmatic subjects showed no significant correlations. There was no significant correlation between peak PM_10 _levels and lung function in either asthmatics or non-asthmatics. In the asthmatic subjects, there were a number of associations noted between pollution and temperature variables and the use of any reliever medication which reached statistical significance. These were daytime indoor pollution – one day lag; night-time indoor pollution – same night and one day lag; temperature; minimum and maximum inside temperature, and relative humidity. There was no significant relationship between the use of any reliever medication and any of the lung function variables in the asthmatic group.

When multivariate analysis was undertaken, using six dependent lung function variables (FEV_1 _and PEF, morning and afternoon and daily variation in both measures), and lagging air pollution measures out to seven days, no pollution variable was retained in a regression model, with only minor temperature effects being retained. When asthmatics were studied separately, small but statistically significant associations between maximum outdoor air pollution levels and variability in FEV_1_, and night time PEFR were noted. With higher maximum out door pollution, FEV1 variability between morning and afternoon dropped, and afternoon PEFR dropped. Effect size was small, in the case of afternoon PEFR having an R^2 ^of 3.6%. Reliever use was not retained in the regression models for any of the lung function data, except for daily variability in PEF.

When comparing median morning FEV_1 _between moderate/high pollution days (>20 μg/m^3^) and low pollution days, students with asthma demonstrated lower readings on high pollution days (p = 0.043). No effect was seen in non-asthmatic students. However, studying moderate/high pollution days only, no significant correlation between lung function and pollution level could be detected, either in asthmatics or normal students.

### Diary card and symptom data

Symptom score data was available for a median of 119 time points (Range 109–119). A number of biologically plausible associations were identified between symptoms and both indoor and outdoor air pollution levels. The proportion of students reporting cough and ear, nose and throat symptoms increased with increasing indoor air pollution the previous day. Maximum outdoor air pollution levels the same day were associated with increasing proportion of students reporting cough. The effect of maximum outdoor air pollution remained significant when multivariate analysis was undertaken, and remained in the model, while weather effects were excluded by the model. The effect size however was small, with a change in PM_10 _of 50 μg/m^3 ^being associated with a 1.5% increase in proportions of children reporting cough (equivalent to one extra report of cough on high pollution days in this cohort). The use of reliever medication was not retained in the model when asthmatics were studied separately, and in this sub-group, the effect of maximum outdoor air pollution remained statistically significant.

## Discussion

This study describes comprehensive pollution and respiratory health monitoring of a panel of students at the boarding house of a metropolitan school. Urinary 1-OHP levels were raised in association with pollution events, but no significant effect of pollution could be seen in the group as a whole either on lung function or airway inflammation and oxidative stress in healthy children. Minor increases in respiratory symptoms such as cough were associated with high pollution exposure. Children with asthma showed some small but statistically significant associations between peak levels of air pollution and lung function and symptoms, but no other significant differences in lung function or biomarkers of airway inflammation could be identified between healthy students and students with asthma.

The importance of accurate assessment of pollution exposure in health effects studies has been recognised since at least the early 1980's. The most accurate measurement tool, personal exposure monitoring, is expensive and logistically difficult, and does not easily lend itself to panel studies involving large numbers of subjects, particularly children. Differing exposures at home compared to work/school also decreases the accuracy of exposure assessment. The effect of cigarette exposure, both personal and via passive smoking, acts as a confounder for the assessment of health effects of air pollution. This study attempted to circumvent these issues by studying schoolchildren living and studying in the same, smoke-free environment. Other potential confounders such as dietary variation might also be expected to be reduced by central institutional catering. The close relationship between indoor and outdoor air pollution levels compares favourably with similar studies which have used personal exposure monitoring [19]. The difference in pollution levels measured at the school, compared with the central monitoring station argues strongly against relying solely on central monitoring for exposure assessment.

A limitation of the approach of studying students in boarding houses of private schools is the potential for decreased generalisability of these results to the rest of the population. Students in this study were all male, predominantly identified themselves as European, and were from wealthier backgrounds than the population of Christchurch generally. It was felt, however, that the advantages of comprehensive pollution and respiratory health monitoring in this panel of non-smoking students out-weighed any potential limitations due to generalisability.

Urinary 1-hydroxypyrene has previously been proposed as a biomarker of exposure to polycyclic aromatic hydrocarbons (PAHs) [20]. Early studies primarily focussed on occupational exposure to PAHs, while recent studies have investigated environmental exposure to PAHs, including via vehicle emissions or indoor sources such as coal burning stoves and tobacco smoke [21,22]. The current study is the first to investigate the utility of 1-OHP for assessing environmental exposure to PAHs suggested to be primarily derived from wood-burning for home-heating. PAH concentrations are highly correlated with particulate air pollution in Christchurch [23]. The potential use of 1-OHP as a biomarker of pollution exposure in this population is explored in more detail elsewhere [6].

Small effects of peak air pollution levels on some lung function measurements were detected in the asthmatic subjects. The effects on afternoon peak flow and decreased variability between morning and evening FEV_1 _might represent a 1/2–1 day lag effect of pollution on lung function in this group. Generally, the effect sizes were small. No significant independent effect of air pollution on lung function was detected in the population as a whole. It is unlikely that reliever use in the asthmatic children significantly reduced any effect size seen, since reliever use was generally very low, and the variable used for any reliever use was only retained in one model in the multivariate analysis. Even attempting to force the variable into the multivariate models did not alter the analyses. The effects in asthmatic students were only seen associated with peak pollution levels, as averaged over a 10 minute period. No association was seen between lung function and 24 hour average pollution levels in the multivariate analyses. This contrasts with the study of Trenga and co-workers who identified a relationship between 24 hour mean pollution levels and lung function in children with asthma [24].

This study also demonstrated small but statistically significant effects of pollution on ear, nose and throat symptoms in this population. This is consistent with previous studies of wood-smoke air pollution [10].

The power of the study may have been reduced by relatively low pollution levels during the winter of 2004, especially during the school terms, when children were collecting lung function data. A systematic review of air pollution and panel studies in children has however suggested publication bias towards adverse effects of particulate air pollution on lung function and lower respiratory symptoms [25].

More recently, researchers have explored the utility of studying airway inflammation in the context of pollution exposure. A number of studies have recently been published describing the relationship of exhaled nitric oxide levels to pollution exposure [26,27]. Nitric oxide was not measured in this current study, due to the lack of portable equipment in 2004. However, exhaled breath condensate has been proposed as another simple non-invasive tool for measurement of airway inflammation [28]. Whilst nitric oxide measurement only provides information about eosinophilic airway inflammation, other markers of airway inflammation such as pH and hydrogen peroxide can be studied in EBC. EBC pH has been shown to vary between individuals and in respiratory conditions such as bronchial asthma, bronchiectasis and chronic obstructive pulmonary disease [29], and EBC H_2_O_2 _is increased in a number of inflammatory conditions such as asthma, chronic obstructive pulmonary disease and community acquired pneumonia [30-33]. Measurement of such "biomarkers of effect" in EBC has been proposed as a way of exploring health effects of air pollution in exposed populations [24].

No demonstrable effect of pollution on EBC pH or H_2_O_2 _was demonstrated in this study, either in healthy children, or children with asthma. The ranges of results for both tests were broad, with no correlations between EBC levels of hydrogen peroxide, and pH, and no correlation with either of these measurements and urine 1-OHP.

Similar issues are described by Doniec and co-workers who studied effects of passive smoking on EBC H_2_O_2 _in 9 year old children [34]. They were unable to demonstrate a difference in EBC H_2_O_2 _between exposed and non-exposed children, though commented that low sensitivity of the assay, and wide spread of measured concentrations of H_2_O_2 _might mask subtle effects on airway inflammation. Similarly, Barregard and co-workers, though they describe statistically significant alterations in EBC malondialdehyde (another proposed biomarker of oxidative stress) after exposure to wood smoke, suggest that the results be interpreted with caution due to very low overall levels in EBC, with many measurements below detection limit of the assay [35]. Assay variability when measuring H_2_O_2 _using fluorimetry and colorimetry is broad in the reported literature, with some studies reporting good agreement of results [36-38], while others show higher coefficients of variation [32,39,40].

EBS pH was measured immediately after collection, without argon degassing or standardisation for CO_2 _partial pressure. There continues to be vigorous debate about the merits of each of these approaches [41], however degassing is not a feasible approach when collecting and processing large numbers of samples in a short period.

The potential drawbacks of EBC analysis, including assay variability and very low biomarker concentration have been highlighted in a recent review article [42] and are addressed in the ATS/ERS taskforce position paper on EBC [43]. At this stage, EBC collection and analysis appears to show utility in highly controlled research settings, but the current collection and analysis techniques are not suitable for application "in the field" for community assessment of effects of air pollution.

## Conclusion

In summary, in this study we detected no significant effect of ambient wood-smoke particulate air pollution on lung function of healthy school-aged male students, but a small effect on cough. Small but significant effects of peak pollution levels were seen in students with asthma. Urinary 1-OHP shows potential as a biomarker of exposure to PAHs in wood smoke in this population; however measurement of EBC pH and hydrogen peroxide using current methods of collection and analysis appears not to be useful for assessment of population health effects of air pollution.

## Competing interests

The authors declare that they have no competing interests.

## Authors' contributions

All authors made substantial contributions to the conception and design of the study, and the acquisition of data. All have been involved in the drafting and revision of the manuscript. All have read and approved the final manuscript. ME was principal investigator for the study, and took overall responsibility for the design, implementation and analysis of the study. SK and TA coordinated the pollution monitoring and analysis and contributed to event study assessments; JC, LB, and KT coordinated the collection and analysis of the urinary 1-OHP samples and contributed to event study assessments; RD, TH, SM, FM, and JC coordinated the diary card and spirometry data acquisition, and coordinated the event study assessments. WB undertook the EBC hydrogen peroxide analysis and contributed to event study assessments. CF was the biostatistician for the study.
